# Genomic data reveals the emergence of an IncQ1 small plasmid carrying *bla*_KPC-2_ in *Escherichia coli* of the pandemic sequence type 648

**DOI:** 10.1016/j.jgar.2021.02.014

**Published:** 2021-06

**Authors:** Bruna Fuga, Louise Cerdeira, Quézia Moura, Herrison Fontana, Danny Fuentes-Castillo, Albalúcia C. Carvalho, Nilton Lincopan

**Affiliations:** a Departmentof Microbiology, Instituto de Ciências Biomédicas, Universidade de São Paulo, São Paulo, Brazil; b Department of Clinical Analysis, Faculdade de Ciências Farmacêuticas, Universidade de São Paulo, São Paulo, Brazil; c One Health Brazilian Resistance Project (OneBR), Brazil; d Department of Infectious Diseases, Central Clinical School, Monash University, Melbourne, Victoria, Australia; e Faculty of Health Sciences, Universidade Federal da Grande Dourados, Dourados, Mato Grosso do Sul, Brazil; f Department of Pathology, Faculdade de Medicina Veterinária e Zootecnia, Universidade de São Paulo, São Paulo, Brazil; g Clinical Laboratory, Universidade Federal da Paraíba, João Pessoa, Paraíba, Brazil

**Keywords:** Enterobacterales, Carbapenemase, KPC-2, Plasmidome, Resistome, Phylogenomics

## Abstract

**Objectives:**

The global success of carbapenem-resistant pathogens has been attributed to large plasmids carrying *bla*_KPC_ genes circulating among high-risk clones. In this study, we sequenced the genome of a carbapenem-resistant *Escherichia coli* strain (Ec351) isolated from a human infection. Phylogenomic analysis based on single nucleotide polymorphisms (SNPs) as well as the comparative resistome and plasmidome of globally disseminated *bla*_KPC-2_-positive *E. coli* strains with identical sequence type (ST) were further investigated.

**Methods:**

Total DNA was sequenced using an Illumina NextSeq 500 platform and was assembled using Unicycler. Genomic data were evaluated through bioinformatics tools available from the Center of Genomic Epidemiology and by in silico analysis.

**Results:**

Genomic analysis revealed the convergence of a wide resistome and virulome in *E. coli* ST648, showing a high-level phylogenetic relationship with a KPC-2-positive ST648 cluster identified in the USA and association with international clade 2. Additionally, the emergence of an IncQ1 small plasmid (pEc351) carrying *bla*_KPC-2_ (on an NTE_KPC_-IId element), *aph(*3'*)-VIa*, and plasmid regulatory and replication genes in the pandemic clone ST648 is reported.

**Conclusion:**

Identification of a *bla*_KPC-2_-positive IncQ1 plasmid in a high-risk *E. coli* clone represents rapid adaptation and expansion of these small plasmids encoding carbapenemases to novel bacterial hosts with global distribution, which deserves continued monitoring.

## Highlights

• Epidemiological success of KPC has been linked to plasmids carrying *bla*_KPC_ genes.
• An IncQ1 small plasmid carrying *bla*_KPC-2_ was found in pandemic *Escherichia coli* ST648.
• Plasmid analysis revealed *bla*_KPC-2_ on an NTE_KPC_-IId element with the *aph(*3'*)-VIa* gene.
• Plasmid phylogeny confirmed >99% identity with IncQ/*bla*_KPC-2_ from *Klebsiella pneumoniae.*
• The emergence and rapid expansion of IncQ1/*bla*_KPC-2_ to novel hosts is discussed.

## 1 Introduction

The epidemiological success of pandemic KPC-type carbapenemases has been linked to plasmids carrying *bla*_KPC_ genes, associated with the mobile element Tn*4401* [1], [2]. The commonest of these is the IncF type, followed by IncN, IncX, IncA/C, IncP, IncL/M, IncR, IncH, IncI, IncU and Col, which have rapidly disseminated, especially among international clones of *Escherichia coli* (ST10, ST38, ST69, ST131, ST155, ST224, ST393, ST405, ST410 and ST648) and *Klebsiella pneumoniae* (most clonal group CG258) species [1], [2], [3], [4], [5].

Plasmids that contain a full set of conjugation genes are called conjugative, whereas small plasmids that contain only a minimal set of genes that allow them to be mobilised by conjugation when they coexist in the same donor cell with a conjugative helper plasmid are called mobilisable plasmids [6]. While conjugative plasmids are generally low copy number, mobilisable small plasmids tend to be high copy number [6]. Small plasmids have begun to be associated with *bla*_KPC_ in members of the *K. pneumoniae* complex and *Klebsiella aerogenes* [7], [8], [9]. Specifically, IncQ1 small plasmids have increased notoriety owing to their broad host range, stability, and ability to harbour genes conferring resistance to clinically relevant antibiotics [7], [8], [9], [10], [11], [12]. In this study, we present genomic data revealing the expansion of IncQ1 small plasmids harbouring the *bla*_KPC-2_ gene to *E. coli* of the pandemic clone ST648.

## 2 Methods

### 2.1 Bacterial strain and antimicrobial susceptibility testing

During a Brazilian surveillance study (OneBR project) conducted to characterise the burden of antimicrobial resistance, a carbapenem-resistant *E. coli* strain (Ec351) was isolated from a blood culture of a patient admitted to the paediatric unit of a university hospital in João Pessoa, Northeastern Brazil. Bacterial identification was performed by matrix-assisted laser desorption/ionisation time-of-flight mass spectrometry (MALDI-TOF/MS) and the antimicrobial susceptibility profile was determined by the disk diffusion method following the recommendations of the Clinical and Laboratory Standards Institute (CLSI supplement M100, 30th ed).

### 2.2 Whole-genome sequencing (WGS)

WGS was carried out on an Illumina NextSeq platform (Illumina Inc., San Diego, CA, USA) using a paired-end library. Briefly, single colonies of bacteria were grown in 3 mL of lysogeny broth for 18 h at 37 °C and the DNA was extracted using a PureLink™ Quick Gel Extraction Kit (Life Technologies, Carlsbad, CA, USA). Total genomic DNA was used for library construction using a Nextera DNA Flex Kit (Illumina Inc.).

### 2.3 Genomic data analysis

The generated raw reads were initially subjected to quality check using FastQC software (http://www.bioinformatics.babraham.ac.uk/projects/fastqc), and the paired reads were trimmed to remove adapters and low-quality regions (Phred quality score, <20) using TrimGalore v.0.6.5 (https://github.com/FelixKrueger/TrimGalore). Subsequently, short-read sequence data were de novo assembled using Unicycler v.0.4.8 (https://github.com/rrwick/Unicycler), being visualised and manually curated using the Bandage assembly graph viewer v.0.8.1 (https://github.com/rrwick/Bandage). Genome annotations were made using NCBI Prokaryotic Genome Annotation Pipeline (PGAP) v.3.2 (http://www.ncbi.nlm.nih.gov/genome/annotation_prok/) and PATRIC v.3.6.5 web server (https://www.patricbrc.org). In silico analyses were performed using ResFinder 4.1, VirulenceFinder 2.0, PlasmidFinder 2.1, MLST 2.0 (Multi-Locus Sequence Typing), SerotypeFinder 2.0 and FimTyper 1.0 online tools from the Center for Genomic Epidemiology (http://www.genomicepidemiology.org), with thresholds set at ≥90% for sequence identity (% ID) and ≥80% for coverage.

The *E. coli* clade was determined by genome-based phylogeny using genomes from *E. coli* ST648 belonging to clades 1, 2, 3 and 4 [13] (ENA accession numbers **ERR1035647,ERR1035691,ERR1035681 andERR279307). The Virulence Factor Database (VFDB)** was also used to evaluate virulence genes (https://github.com/haruosuz/vfdb). The *E. coli* phylogroup was determined by ClermonTyping v.1.4.0 (https://github.com/A-BN/ClermonTyping). ABRicate v.0.9.8 (https://github.com/tseemann/abricate) was used to predict heavy metal (arsenic, lead and mercury) and disinfectant (quaternary ammonium compounds) resistance genes using the BacMet2 database (http://bacmet.biomedicine.gu.se/). Additionally, pesticide (glyphosate) resistance genes were identified by in silico comparative analysis against an in-house database. For all predicted genes, a >90% identity threshold was used as filter for identification.

The *bla*_KPC-2_-containing plasmid was mapped using paired-end short reads to close gaps and was manually curated based on sequence homology to the reference IncQ1 plasmid pKPN535a (GenBank accession no. **MH595533)**. Mauve alignment generated using Geneious software, and BLAST Ring Image Generator (BRIG) (https://github.com/happykhan/BRIG) were used for plasmid comparison and design. IncQ1/*bla*_KPC-2_ plasmids previously identified in *K. pneumoniae* of CG258 (pKPN535a, GenBank accession no. **MH595533; pKPC05, GenBank accession no.MK330868; and pB29, GenBank accession no.MK330869), Klebsiella***quasipneumoniae* (pKQPS142b; GenBank accession no. **CP023480.1)** and *K. aerogenes* (pEa33A; GenBank accession no. **MH000708) strains** were included in the comparative analysis.

The phylogenetic relationship based on single nucleotide polymorphism (SNP) analysis as well as the comparative resistome and plasmidome of KPC-2-positive *E. coli* ST648 strains from the USA (*n* = 15), Colombia (*n* = 1), Brazil (*n* = 1) and Greece (*n* = 2) were investigated using publicly available genomes deposited in the GenBank database (https://www.ncbi.nlm.nih.gov/genbank/) (Supplementary Table S1), along with the genome sequenced in the current study. Annotated draft assemblies produced by Prokka (https://github.com/tseemann/prokka) were employed in pangenome pipeline Roary v.3.13.0 (https://github.com/sanger-pathogens/Roary). SNPs were extracted from the core gene alignment using SNP-sites (https://github.com/sanger-pathogens/snp-sites), and a maximum-likelihood tree based on SNP alignment was constructed using RAxML-NG v.0.9.0 (https://github.com/amkozlov/raxml-ng), under the generalised time-reversible model with gamma-distributed rate heterogeneity. The phylogeny was tested against 1000 bootstrap replications and the resulting tree was visualised with iTOL v.5.6.1 (https://itol.embl.de/).

## 3 Results

*E. coli* Ec351 strain exhibited a multidrug-resistant profile to ampicillin, aztreonam, amoxicillin/clavulanic acid, cefalotin, ceftriaxone, cefotaxime, cefoxitin, ceftazidime, cefepime, ertapenem, meropenem, imipenem, nalidixic acid, ciprofloxacin, trimethoprim/sulfamethoxazole, amikacin and tetracycline, but remained susceptible to gentamicin, chloramphenicol and fosfomycin.

Trimmed paired-end reads were assembled into 246 contigs with a mean coverage of 180×. WGS analysis revealed a genome size of 5 318 322 bp with a GC content of 50.3%, an *N*_50_ value of 101 142, and 5049 protein-coding genes, 48 RNA-coding genes and 103 pseudogenes. The circular plot and subsystems obtained from PATRIC (https://www.patricbrc.org/) are shown in Supplementary Fig. S1A and S1B, respectively. In this regard, PATRIC functional annotation revealed that the genome of Ec351 has 989 genes for metabolism, 268 genes for protein processing, 117 genes involved in DNA metabolism, and 205 genes for virulence, stress response and defence.

Epidemiological analysis based on MLST, Clermont typing and phylogenomics revealed that strain Ec351 belonged to ST648 (displaying >70% identity with clade 2; Supplementary Table S1) and phylogroup F. This sequence type is the primary founder of CG648 recognised as an international multiresistant high-risk clone [3], [13], [14]. In this regard, *E. coli* ST648 has contributed to the dissemination of *bla*_KPC_ genes in China, Greece and the USA [15], [16], [17], [18]. In South America, KPC-positive *E. coli* ST648 have been reported in Colombia [1] and Brazil [19].

**Fig. 1:**
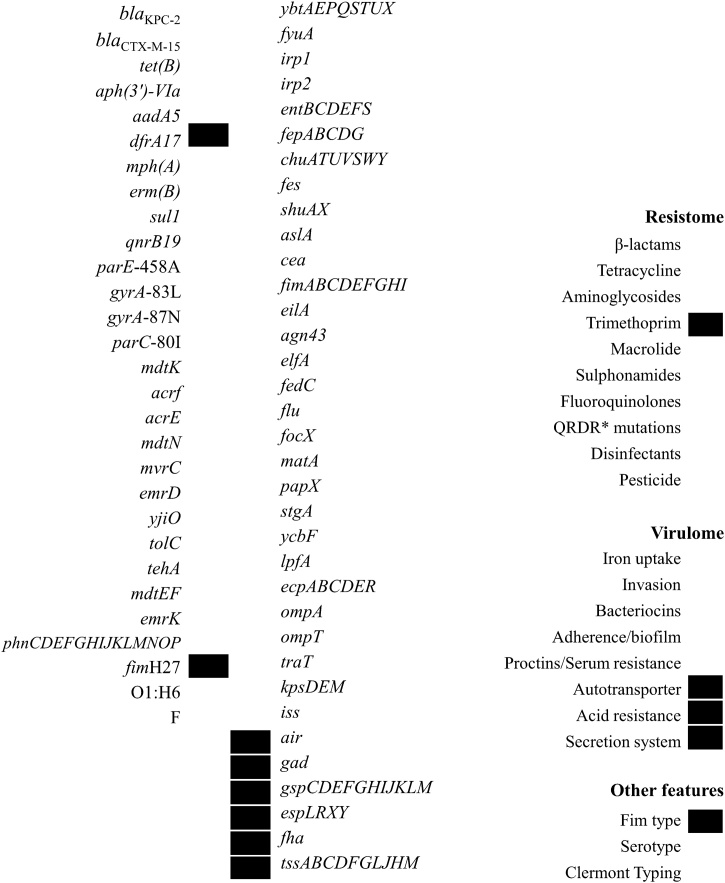
Resistome, virulome, Clermont typing, *fim* type and serotype of clinical *Escherichia coli* ST648 strain Ec351. QRDR, quinolone resistance-determining region.

The convergence of a wide virulome (adherence, biofilm, autotransporter, iron uptake, invasion, bacteriocins, serum resistance, acid resistance and secretion system genes) and resistome (antibiotic, disinfectant and pesticide resistance genes) was confirmed in Ec351 (Fig. 1) and must contribute to the clinical success of ST648. Additionally, IncFIA, IncFIB-like, IncFII-like, IncX4 and IncQ1 plasmid incompatibility groups were predicted.

**Fig. 2:**
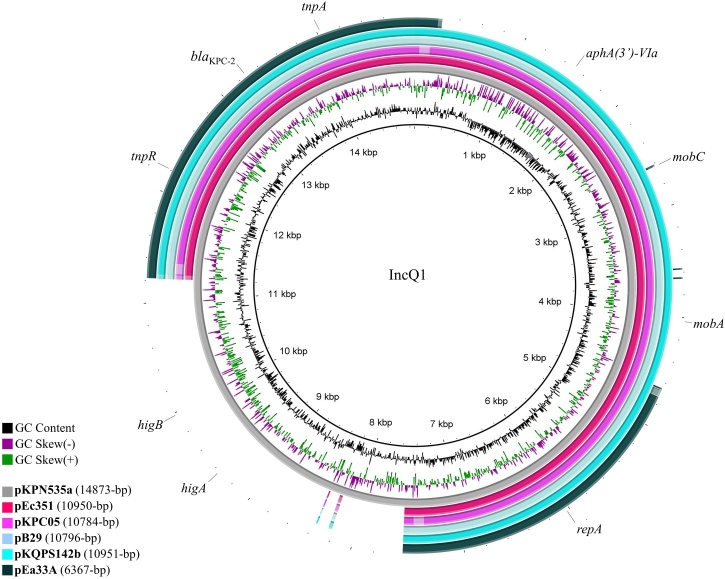
Comparison of *bla*_KPC-2_-positive IncQ1 plasmids identified in *Escherichia coli* Ec351 belonging to ST648 (pEc351, GenBank accession no. **MT349421), Klebsiella***pneumoniae* of CG258 [GenBank accession numbers **MH595533 (pKPN535a),MK330868 (pKPC05) andMK330869 (pB29)], Klebsiella***quasipneumoniae* [GenBank accession no. **CP023480.1 (pKQPS142b)] and Klebsiella***aerogenes* [GenBank accession no. **MH000708**(pEa33A)] strains. Uncoloured lines indicate different regions among the plasmids. The genetic structure of plasmid pEc351 included *bla*_KPC-2_ and *aph(*3'*)-VIa* genes encoding resistance to carbapenems and aminoglycosides, respectively, along with replication and mobilisation modules (*repA*, *mobA* and *mobC*) and accessory genes (*tnpR* and *tnpA*).

The *bla*_KPC-2_ gene was located in a NTE_KPC_-IId element [8] on the IncQ1 plasmid, named pEc351 (GenBank accession no. **MT349421**). This plasmid is ∼10.1kb in size, sharing high nucleotide identity (>99%) with overlapping regions of IncQ1/*bla*_KPC-2_ plasmids previously identified in *K. pneumoniae* of CG258 (pKPN535a, pKPC05 and pB29), *K. quasipneumoniae* (pKQPS142b) and *K. aerogenes* (pEa33A) strains (Fig. 2). The genetic structure of the pEc351 plasmid also included the *aph(*3'*)-VIa* gene encoding resistance to aminoglycosides, along with replication and mobilisation modules (*repA*, *mobA* and *mobC*) and accessory genes (*tnpR* and *tnpA*). Comparative plasmid sequence analysis of IncQ1/*bla*_KPC-2_ plasmids showed that *aph(3′)-Vla*, *mobA* and *mobC* were not present in pEa33A, whereas genes encoding HigB–HigA toxin/antitoxin complex and DNA-binding transcriptional repressor were absent in pEc351, pKPC05, pB29, pKQPS142b and pEa33A plasmids compared with the reference plasmid pKPN535a (Supplementary Fig. S2). Interestingly, IncQ1/*bla*_KPC-2_-positive *Klebsiella* spp. and *E. coli* Ec351 strains co-carried broad-host-range IncF vectors that can efficiently mobilise non-conjugative small plasmids [2]. Although strain Ec351 carried IncF and IncX4 plasmids, considering the limitations of short-read sequencing, it was only possible to assemble the IncX4 plasmid (pEcIncX4; GenBank accession no. **MT349420**). In this regard, although pEcIncX4 did not carry resistance genes, the presence of *trbM*, *traG*, *virB11*, *virB10*, *virB9*, *virB8*, *virB6*, *virB5*, *virB4* and *virB2* genes encoding components of the conjugative transfer apparatus as well as *hicB* and *hicA* type II toxin/antitoxin system genes was confirmed, supporting the hypothesis that IncX4 could contribute to the mobilisation of the IncQ1/*bla*_KPC-2_ plasmid pEc351 [20].

**Fig. 3:**
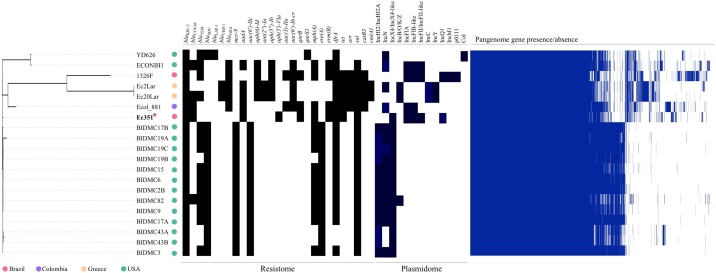
Maximum-likelihood phylogenetic tree constructed based on the core genome of KPC-2-positive *Escherichia coli* ST648 lineages. The image was visualised using iTOL v.5.6.1 (https://itol.embl.de). On the right, the panel represents the resistome and plasmidome of *E. coli* genomes. Light blue regions in the plasmidome represent the Inc-type plasmid carrying the *bla*_KPC-2_ gene. For some *E. coli* isolates from the USA (YD626, BIDMC17B, BIDMC19B, BIDMC6, BIDMC2B, BIDMC82, BIDMC9, BIDMC17A and BIDMC3) and Brazil (1326F), it was not possible to confirm the plasmid Inc group carrying the *bla*_KPC-2_ gene. Subsequently, the panel shows pangenome variation based on core and accessory genome components (gene presence/absence matrix), which was graphically represented using the roary_plots.py script.

Based on core genome SNPs, phylogenetic analyses of KPC-2-positive *E. coli* strains belonging to the international ST648 analysed 3978 genes shared by all isolates. In this regard, the phylogenetic tree highlights the high relationship (>95% identity; Supplementary Table S2) between Ec351 and the USA cluster formed by 13 KPC-2-positive isolates co-carrying the *mcr-9* colistin resistance gene (Fig. 3). Interestingly, Ec351 and the USA cluster exhibit an identical resistome and plasmidome, associated with *bla*_KPC-2_ (β-lactam resistance), *mphA* (macrolide resistance), *dfrA* (trimethoprim resistance) and *sul1* (sulfonamide resistance) genes and IncX4-type plasmids, respectively (Fig. 3; Supplementary Table S3). On the other hand, bioinformatics analysis of the publicly available genome of a *bla*_KPC-2_-positive *E. coli* ST648 (strain 1325F) isolated in 2013 from a rectal swab of a patient in Southern Brazil identified IncQ1 along with IncF-type plasmids, supporting the hypothesis of early dissemination of this clone and the stability of small IncQ1 plasmids carrying *bla*_KPC-2_ in this country. On the other hand, while IncHI2/IncHI2A, IncN and IncC plasmids were most associated with *bla*_KPC-2_ genes among *E. coli* ST648 from the USA, Colombia and Greece, the IncQ1 plasmid harbouring *bla*_KPC-2_ was only identified in Brazil (Fig. 3). For some *E. coli* isolates from the USA (YD626, BIDMC17B, BIDMC19B, BIDMC6, BIDMC2B, BIDMC82, BIDMC9, BIDMC17A and BIDMC3) and Brazil (1326F), it was not possible to confirm the plasmid Inc group carrying the *bla*_KPC-2_ gene.

## 4 Discussion

Carbapenem-resistant Enterobacterales have been recognised as critical priority pathogens by the World Health Organization (WHO) (https://www.who.int). In this study, the complete sequence of an IncQ1 plasmid carrying the *bla*_KPC-2_ gene is reported for the first time in an international clone of *E. coli* ST648. In this respect, while mobilisation of the *bla*_KPC-2_ gene among *E. coli* strains belonging to ST648 has been mediated by IncN and IncHI2 large plasmids [1], [15], [19], IncQ1 *bla*_KPC-2_-positive plasmids have been identified in *K. pneumoniae* of CG258, *K. quasipneumoniae*, *K. aerogenes* and *Pseudomonas aeruginosa* so far [7], [8], [9], [11], [12], [21], denoting an emerging mechanism responsible for the dissemination of carbapenem resistance that may carry a lower fitness cost and could potentially contribute to increased prevalence and persistence [7], [8], [9], [22]. In fact, bacterial cells carrying small plasmids can gain an immediate fitness advantage from reducing the burdensome expression of accessory genes, whereas antibiotic resistance could be enhanced by increases in plasmid copy number [4], [23]. In this regard, small plasmids (<20 kb) of the IncQ family present a high copy number (∼10–12 copies/cell or more) in *E. coli* and *P. aeruginosa*, being highly promiscuous due to their ability to be mobilised very efficiently by self-transmissible plasmids [10]. Therefore, these multicopy plasmids could potentiate the evolution of antibiotic resistance in clinically important bacteria [23].

Currently, IncQ-type plasmids have begun to be increasingly identified as carriers of clinically relevant antibiotic resistance genes in human and veterinary isolates [7], [8], [9], [10], [19], being increasingly associated with β-lactam resistance determinants (e.g. *bla*_CMY_-, *bla*_GES_-, *bla*_IMP_- and *bla*_BKC_-type genes) in Enterobacterales such as *E. coli*, *K. pneumoniae* and *Salmonella enterica* [2], [24]. More recently, tigecycline resistance gene *tet*(X4)-bearing IncQ1 plasmids have been identified in *E. coli* from human and non-human hosts [25]. Interestingly, during analysis of publicly available *E. coli* genomic data, we noticed that a genome containing the *bla*_KPC-2_ gene and the IncQ1 plasmid from Brazil has been recently submitted to GenBank (accession no. **LNZU00000000.1)**. Although no additional information is available, the possibility that IncQ1/*bla*_KPC-2_-positive *E. coli* have spread in Brazil is deeply concerning.

## 5 Conclusions

The identification of *bla*_KPC-2_-positive IncQ1 plasmids in a high-risk clone of *E. coli* indicates rapid adaptation and expansion of these small plasmids encoding carbapenemases to novel bacterial hosts with global distribution, which deserves continued monitoring. Finally, this study may give a genomic insight into the spread of WHO critical priority pathogens in a One Health context.

## Data availability

This Whole Genome Shotgun project has been deposited at DDBJ/ENA/GenBank under the accession no. JAANTC000000000 (SRA no. SRR12248448). The version described in this paper is version JAANTC000000000.1. Additionally, genomic data of *E. coli* Ec351 is available on the OneBR platform under the number ID ONE16 (http://onehealthbr.com/).

## Funding

This study was funded by the Bill and Melinda Gates Foundation, Grand Challenges Explorations Brazil–New approaches to characterize the global burden of antimicrobial resistance [grant OPP1193112], Conselho Nacional de Desenvolvimento Científico e Tecnológico (CNPq) [grants AMR443819/2018-1,433128/2018-6 and312249/2017-9] and Coordenação de Aperfeiçoamento de Pessoal de Nível Superior [grants 88887.358057/2019-00 and1794306]. NL is a research fellow of CNPq [grant 312249/2017-9]. The funding sources played no role in the study design, the collection, analysis and interpretation of data, or in writing the manuscript.

## Competing interests

None declared.

## Ethical approval

Not required.
