# Supplementary material for: Genomic data reveals the emergence of an IncQ1 small plasmid carrying blaKPC-2 in Escherichia coli of the pandemic sequence type 648
Source: J Glob Antimicrob Resist. 2021 Jun;25:8–13. doi: 10.1016/j.jgar.2021.02.014 (PMC8213540; doi:10.1016/j.jgar.2021.02.014)
Supplement: Supplementary file 1 [file mmc1.pdf]

A

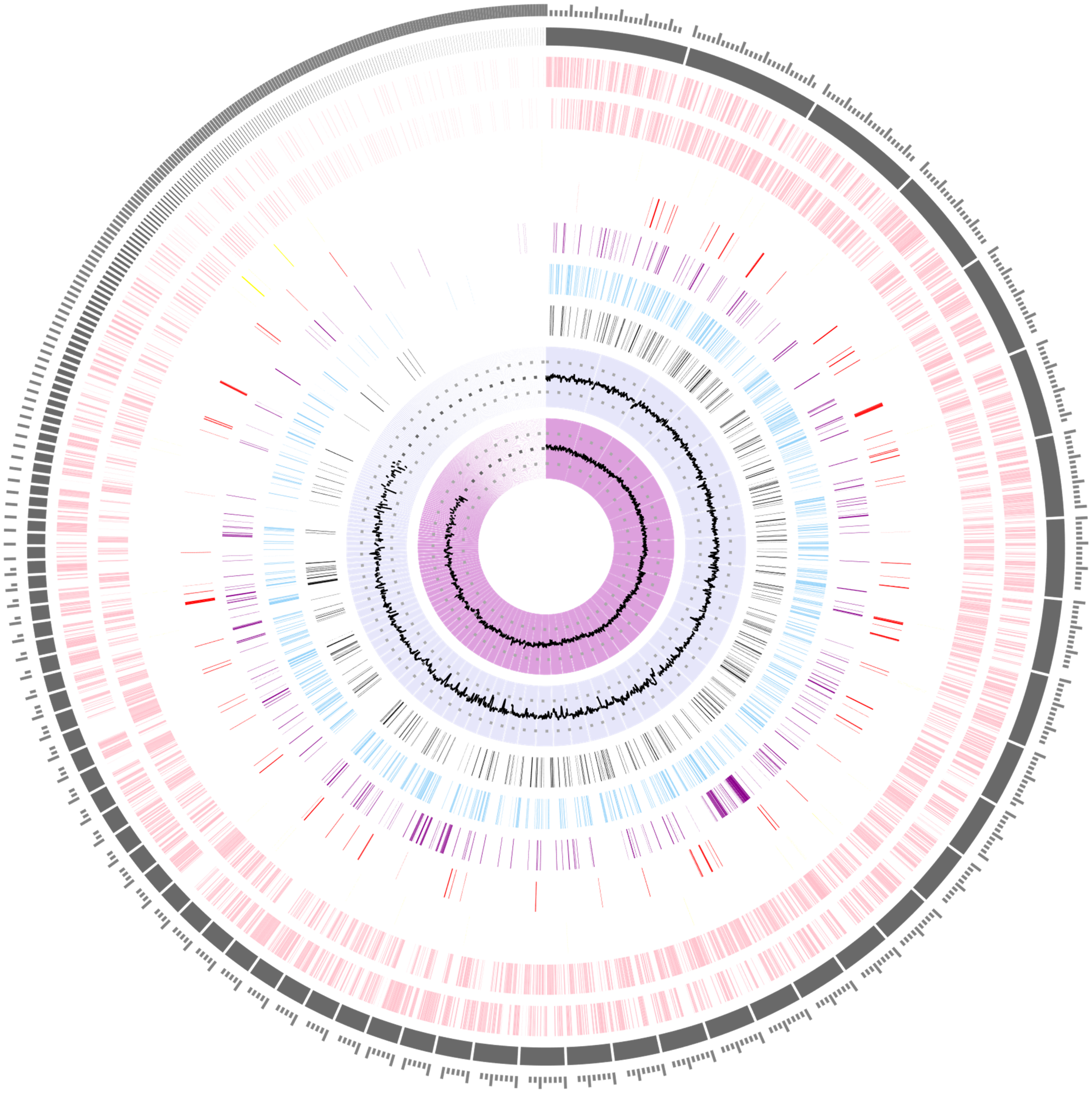

B

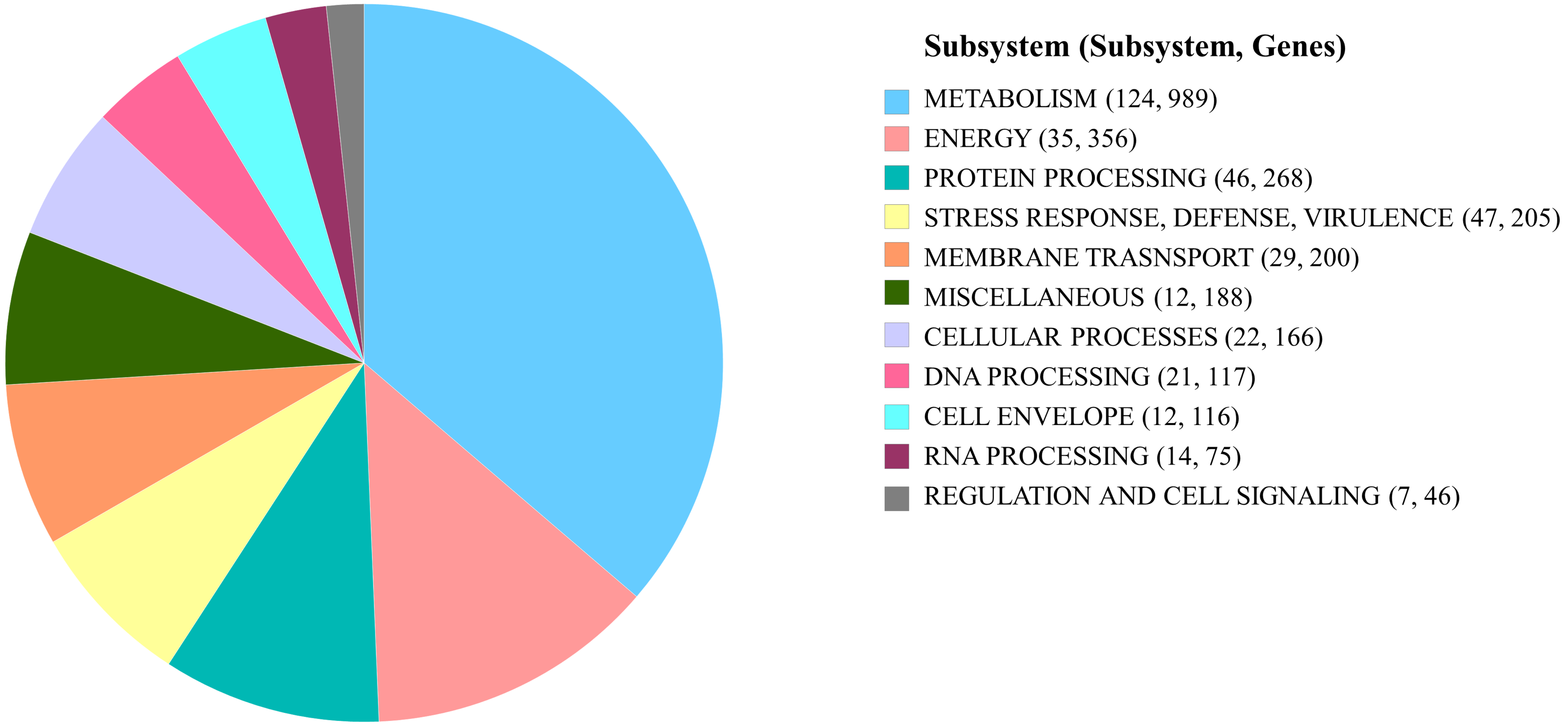
Subsystem (Subsystem, Genes)
METABOLISM (124, 989)
ENERGY (35, 356)
PROTEIN PROCESSING (46, 268)
STRESS RESPONSE, DEFENSE, VIRULENCE (47, 205)
MEMBRANE TRASNSPORT (29, 200)
MISCELLANEOUS (12, 188)
CELLULAR PROCESSES (22, 166)
DNA PROCESSING (21, 117)
CELL ENVELOPE (12, 116)
RNA PROCESSING (14, 75)
REGULATION AND CELL SIGNALING (7, 46)
