# Supplementary figures and images for: Genomic data reveals the emergence of an IncQ1 small plasmid carrying blaKPC-2 in Escherichia coli of the pandemic sequence type 648
Source: J Glob Antimicrob Resist. 2021 Jun;25:8–13. doi: 10.1016/j.jgar.2021.02.014 (PMC8213540; doi:10.1016/j.jgar.2021.02.014)

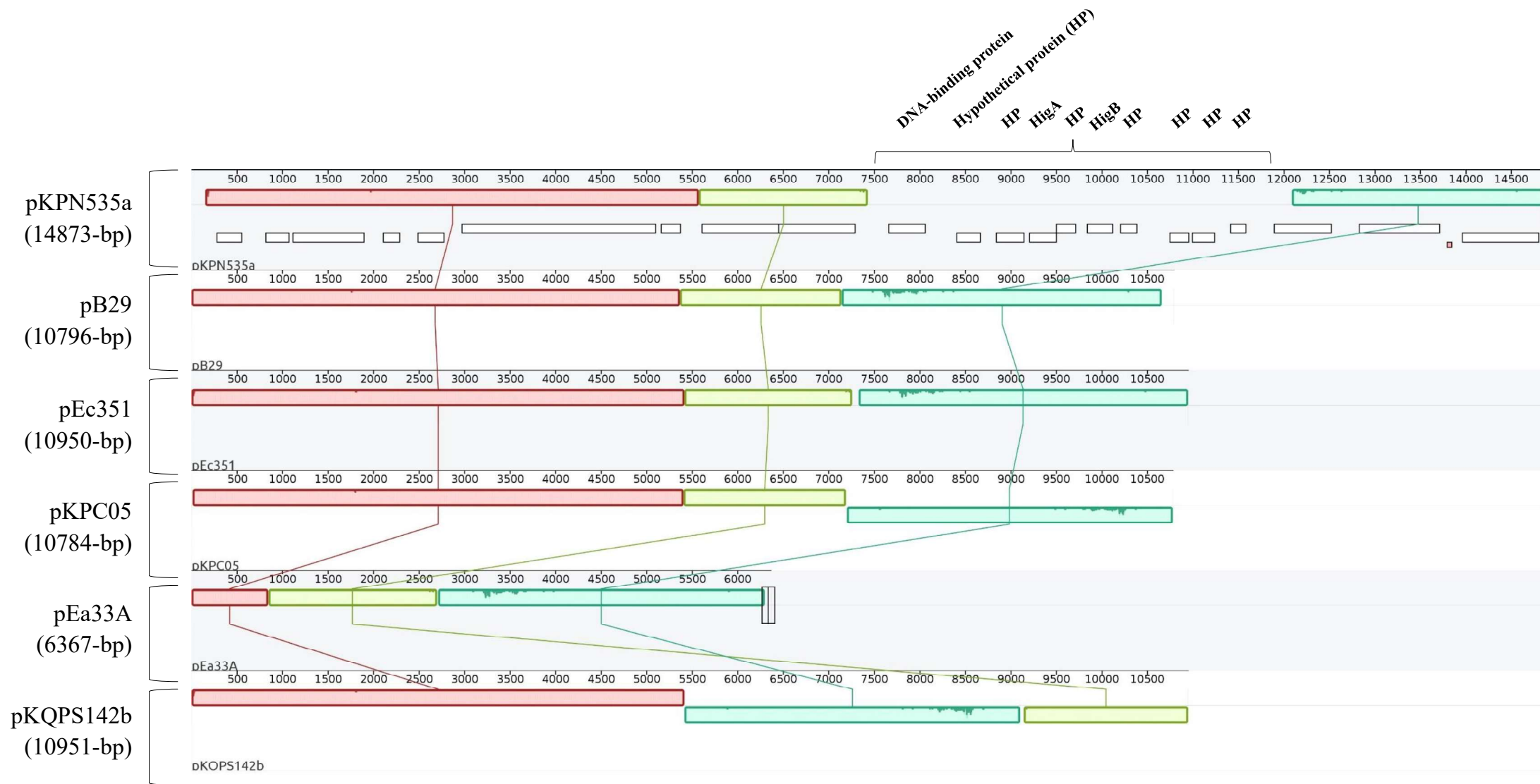

Supplement: Supplementary file 2 [file mmc2.pdf]
