# Supplementary material for: Genomic data reveals the emergence of an IncQ1 small plasmid carrying blaKPC-2 in Escherichia coli of the pandemic sequence type 648
Source: J Glob Antimicrob Resist. 2021 Jun;25:8–13. doi: 10.1016/j.jgar.2021.02.014 (PMC8213540; doi:10.1016/j.jgar.2021.02.014)
Supplement: Supplementary file 6 [file mmc6.docx]

**Supplementary Information**

**Table S1.** Data obtained from genome-based phylogeny analysis of *E. coli* strains of ST648 belonging to clades 1, 2, 3, and 4, and *E. coli* Ec351.

**Table S2.** Data obtained from phylogenetic analyses of globally disseminated *bla*_KPC-2_-positive *Escherichia coli* strains belonging to ST648.

**Table S3**. Epidemiological data, accession numbers, and resitome and plasmidome of globally disseminated *bla*_KPC-2_-positive *Escherichia coli* strains belongingto ST648.

**Figure S1.** In **A**, circular graphical display of the distribution of the contigs of genome assembly. Genome annotations includes (from outer to inner rings): the contigs (dark grey), CDS on the forward strand, CDS on the reverse strand (both in light pink), non-CDS features (light yellow), CDS with homology to known antimicrobial resistance genes (red), CDS with homology to know virulence factors (purple), transporters (blue), drug targets (black), GC content and GC skew. In **B**, PATRIC functional annotation of *E. coli* Ec351 strain belonging to ST648. Pie chart shows functional annotation of several subsystem genes.

**Figure S2**. Mauve comparison of *bla*_KPC-2_-positive IncQ1 plasmids from *E. coli*Ec351 belonging to ST648 (pEc351; GenBank accession number: MT349421), *K. pneumoniae* (pKPN535a, pKPC05, pB29; GenBank accession numbers: MH595533, MK330868 and MK330869), *K. quasipneumoniae* (pKQPS142b; GenBank accession number: CP023480.1), and *K. aerogenes* (pEa33A; GenBank accession number: MH000708) strains. The colored boxes represent syntenic segments connected by lines between genomes, while regions outside blocks lack homology between genomes.
